# XELOX combined with sintilimab and hyperbaric oxygen therapy for advanced or metastatic gastric/gastroesophageal junction adenocarcinoma: study protocol for a prospective, single-arm, phase Ib/II clinical trial

**DOI:** 10.3389/fimmu.2025.1672725

**Published:** 2026-01-12

**Authors:** Wenke Li, Pengfei Zhang, Mo Cheng, Jing Wei, Menghui Xu, Dan Li, Sihui Song, Ming Liu, Cheng Huang, Lin Zhu

**Affiliations:** 1 Gastric Cancer Center/Cancer Center, West China Hospital, Sichuan University, Chengdu, Sichuan, China; 2 Department of Rehabilitation Medicine, West China Hospital, Sichuan University, Chengdu, Sichuan, China; 3 Department of Integrated Traditional and Western Medicine, West China Hospital, Sichuan University, Chengdu, China

**Keywords:** gastric neoplasms, hyperbaric oxygenation, immunotherapy, clinical trial, protocol

## Abstract

**Background:**

Gastric and gastroesophageal junction cancer (GC/GEJC) is the fifth most common and deadliest cancers worldwide, with five-year survival rates ranging from 20–40% due to late-stage diagnosis. First-line treatment for HER2-negative advanced or metastatic GC/GEJC involves chemotherapy combined with PD-1 inhibitors, achieving an objective response rate (ORR) of approximately 60%. However, primary and acquired resistance limits effectiveness, highlighting the need for novel strategies. Tumor hypoxia reduces the efficacy of immune checkpoint inhibitors (ICIs). Hyperbaric oxygen therapy (HBOT) may alleviate hypoxia, enhance drug delivery, and improve immune cell infiltration, potentially increasing the antitumor effects of ICIs.

**Methods:**

This prospective, single-center, single-arm phase Ib/II trial evaluated the efficacy and safety of the XELOX regimen combined with sintilimab and HBOT in HER2-negative advanced or metastatic GC/GEJC patients. Phase Ib employs a 3 + 3 dose-escalation design with nine patients to assess safety and determine the optimal HBOT protocol. Phase II will enrol 48 patients, accounting for a 5% dropout rate, with a focus on the ORR as the primary endpoint. The secondary endpoints include progression-free survival (PFS), the disease control rate (DCR), 2-year disease free survival (DFS), two-year overall survival (OS), quality of life (QoL), and safety. All participants received XELOX, sintilimab and HBOT. Efficacy is assessed every two cycles, with maintenance therapy continuing until disease progression or other termination criteria are met.

**Discussion:**

This is the first clinical trial to explore the efficacy and safety of HBOT combined with chemotherapy and immunotherapy in HER2-negative advanced or metastatic GC/GEJC patients. These results may provide a novel treatment strategy for patients with advanced GC/GEJC, addressing the current limitations of immunotherapy resistance.

**Clinical Trial Registration:**

ClinicalTrials.gov, identifier NCT06742411.

## Trial registration details

**Figure 1:**
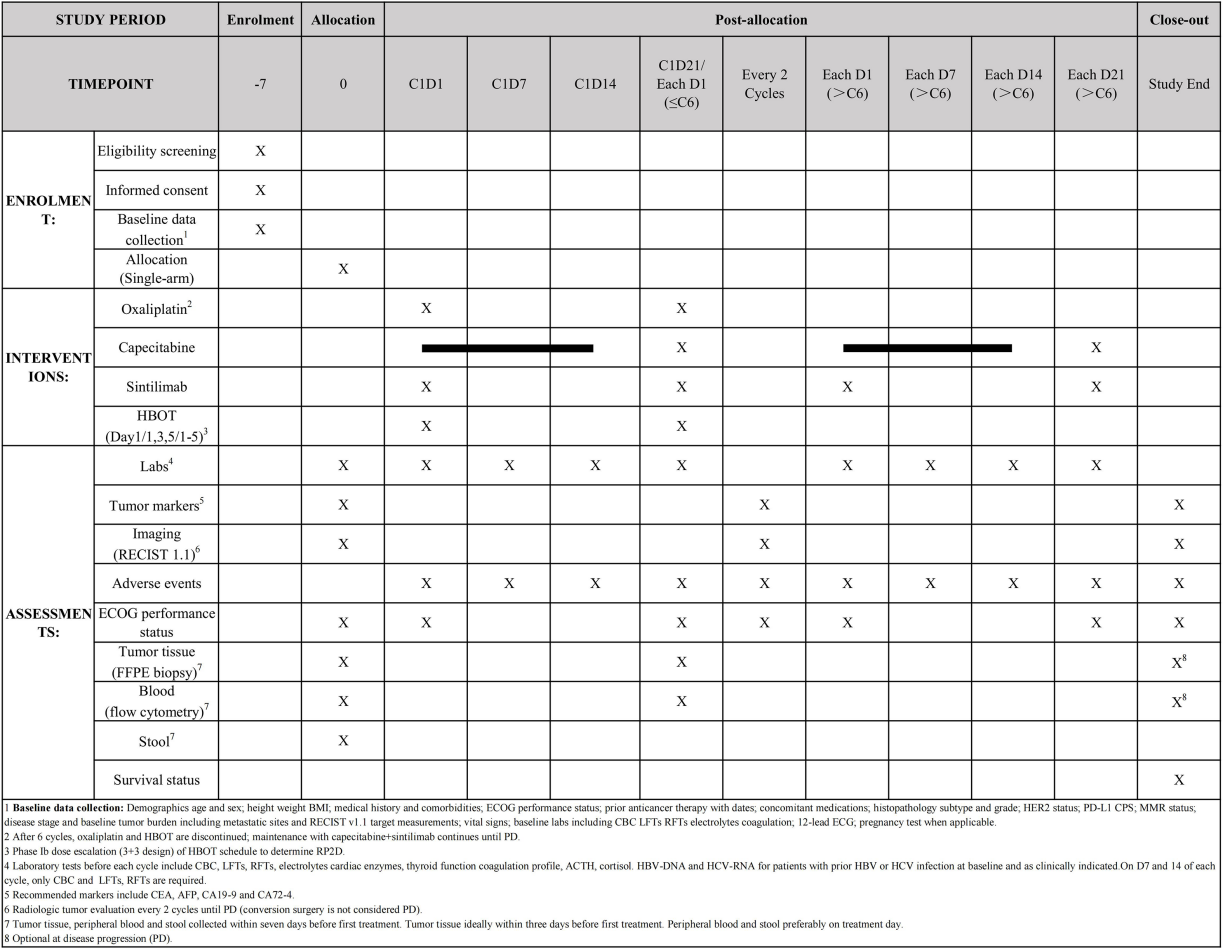
SPIRIT figure. SPIRIT showing the schedule of enrollment, interventions, and assessments in the Phase Ib/II single-arm trial evaluating XELOX, sintilimab, and HBOT in patients with HER2-negative advanced or metastatic GC/GEJC. The timeline encompasses initial screening procedures and baseline data collection, followed by six 21-day cycles of therapy in which oxaliplatin and sintilimab are administered intravenously on Day 1 of each cycle, capecitabine is given orally on Days 1–14 of each cycle, and HBOT at 2.0 atmospheres absolute for 60 minutes is delivered on Day 1 after the drug infusions. After completing six cycles, patients transition to a maintenance phase with capecitabine and sintilimab only. Radiologic tumor evaluations are performed every two cycles until disease progression, and biomarker specimens are collected at predefined time points, including an optional tumor biopsy at progression. Throughout the study, each cycle includes pre-treatment laboratory tests, tumor marker measurements, and ECOG performance status assessments, and all adverse events are closely monitored.

The clinical trial is registered at ClinicalTrials.gov (Identifier: NCT06742411) and conforms to the World Health Organization Trial Registration Data Set. The schedule of enrolment, interventions, and assessments is illustrated in **Figure 1**. This study is conducted in accordance with Protocol Version 1.3, dated 31 October 2024. Participant recruitment began on 31 December 2024 and is expected to be completed by approximately 31 December 2026.

## Introduction

Gastric cancer (GC) is the fifth most common and deadly malignancy worldwide. In 2022, there were approximately 960,000 new cases of GC/GEJC globally, resulting in over 650,000 deaths (1). The primary treatment for GC involves surgical intervention via a multidisciplinary approach. However, owing to its often insidious onset, most patients are diagnosed at a stage with distant metastasis, leading to an overall poor prognosis, with five-year survival rates ranging from 20% to 40% (2). For these patients, chemotherapy-based medical treatment remains the main therapeutic strategy. In recent years, immune checkpoint inhibitors (ICIs) have been successfully applied in various cancers. In the treatment of GC/GEJC, several phase III clinical trials, including CheckMate-649, Orient 16, and Rationale 305, have demonstrated that, compared with chemotherapy alone, combining PD-1 inhibitors with chemotherapy significantly improves progression-free survival (PFS) and overall survival (OS) (3–5). On the basis of these findings, multiple guidelines recommend chemotherapy combined with PD-1 inhibitors as first-line treatment for HER2-negative advanced or metastatic GC. However, the objective response rate (ORR) for PD-1 inhibitors combined with chemotherapy is only approximately 60%, indicating that many GC/GEJC patients do not benefit from PD-1 inhibitor therapy because of primary resistance (3). Additionally, some patients who initially respond may experience relapse, reflecting acquired resistance (6). Therefore, there is an urgent need for new therapeutic strategies in clinical practice to further increase the efficacy of immunotherapy.

Hypoxia is a common feature of most solid tumors and is one of the mechanisms limiting the efficacy of ICIs within the hypoxic tumor microenvironment (7). Hypoxia activates the hypoxia-inducible factor-1 (HIF-1) signaling pathway, increasing the expression of collagen genes and connective tissue growth factor (CTGF), thereby promoting collagen synthesis and the formation of a dense extracellular matrix (ECM) (8). The abnormal ECM and inherent stress generated by tumor growth compress the tumor’s blood and lymphatic vessels, reducing tumor perfusion and impairing drug delivery to the tumor tissue. This creates a physical barrier against ICIs and cytotoxic T lymphocytes (CTLs), thereby diminishing the efficacy of ICIs (9, 10). In addition to causing an abnormal ECM, hypoxia affects CTL function by cultivating an immunosuppressive microenvironment through various pathways. First, hypoxia directly upregulates the expression of programmed death-ligand 1 (PD-L1), leading to the depletion and inhibition of CTL function (11). The hypoxia/HIF-1 axis also promotes the recruitment and accumulation of immunosuppressive regulatory T (Treg) cells, myeloid-derived suppressor cells (MDSCs), and M2 phenotype tumor-associated macrophages (TAMs) (12, 13). These immunosuppressive cells further secrete inhibitory cytokines, collectively coordinating an immunosuppressive microenvironment. Cancer stem cells (CSCs), which are highly proliferative, possess self-renewal capabilities, and have multilineage differentiation potential, have been shown to play significant roles in tumor multidrug resistance, angiogenesis, and invasive metastasis (14, 15). Previous studies have indicated that hypoxia can upregulate the expression of various CSC-related signaling pathway molecules, including Notch, Wnt, and Sonic hedgehog (Shh), and increase the expression of CSC-like proteins (16, 17). Therefore, overcoming tumor hypoxia is crucial for the effective utilization of immune checkpoint blockade (ICB) antibody-mediated immunotherapy.

Hyperbaric oxygen therapy (HBOT), which involves the inhalation of pure oxygen under high pressure, can significantly increase oxygen levels in the blood and tissues and has been widely used to treat various hypoxia-related diseases. HBOT can effectively alleviate tumor hypoxia and is a potent means of reversing the hypoxic tumor microenvironment. Studies have shown that HBOT can reduce collagen deposition through the hypoxia/HIF-1α/CTGF/type I collagen signaling axis and degrade the ECM by generating excessive reactive oxygen species (ROS), thereby promoting drug delivery and CTL infiltration (18–20). Additionally, HBOT can reduce the recruitment of immunosuppressive cells by decreasing the activation of the HIF-1α pathway (18, 21, 22). Moreover, a high-oxygen environment increases the expression of the platelet endothelial cell adhesion molecule CD31, promoting vascular normalization and increasing immune cell infiltration into tumor sites (23). Combining HBOT with chemotherapy can enhance the efficacy of PD-1 antibodies by activating the cGAS-STING signaling pathway, thereby increasing the function of effector T cells and natural killer cells (24). These data suggest that HBOT can significantly increase the efficacy of ICIs and may represent a promising strategy for advanced GC/GEJC.

In this study, we investigated the efficacy and safety of XELOX combined with sintilimab and HBOT in HER2-negative advanced or metastatic GC/GEJC.

## Methods

This is a prospective, single-center, single-arm phase Ib/II clinical trial designed to evaluate the efficacy and safety of XELOX in combination with sintilimab and HBOT in HER2-negative advanced or metastatic GC/GEJC patients. The study protocol and informed consent forms were reviewed and approved by the Academic Review Committee and Ethics Committee of West China Hospital, Sichuan University (WCH20242269). The clinical trial will be conducted in accordance with the Declaration of Helsinki and good clinical practice guidelines. All the participants provided written informed consent, and all the data were kept confidential. No independent committees were established for this single-arm clinical trial. The clinical trial is prospectively registered on ClinicalTrials.gov (NCT06742411).

Phase Ib uses a standard 3 + 3 dose−escalation design to evaluate the safety/tolerability of XELOX plus sintilimab with HBOT and to identify the recommended Phase II HBOT regimen (RP2D). Approximately nine patients are expected in Phase Ib per the 3 + 3 algorithm (no formal power calculation), and Phase Ib enrollment does not contribute to the Phase II power determination.

For Phase II, the sample size is based on the primary endpoint of ORR. Historical first−line data for XELOX plus PD−1 inhibitors suggest an ORR of 58%. Assuming the addition of HBOT increases ORR to 73%, a two−sided α=0.20 and 80% power in an exact single−arm binomial framework yield a required sample size of 45 patients. Allowing for 5% attrition, the planned Phase II enrollment is 48 patients. This calculation is signal−seeking and estimation−oriented rather than confirmatory. Detailed statistical methods are described in the Statistical Analysis section.

### Endpoints

The primary goal of Phase Ib is to assess the safety and tolerability of the XELOX regimen combined with sintilimab and HBOT in treating advanced or metastatic GC/GEJ adenocarcinoma and to determine the optimal HBOT protocol for Phase II.

The primary endpoint of Phase II is the objective response rate (ORR). The secondary endpoints include PFS, DCR, two-year DFS (for patients converted to resectable disease and achieving R0 resection), two-year OS, and quality of life and safety profiles. Adverse events (AEs) will be recorded and evaluated according to the National Cancer Institute Common Terminology Criteria for Adverse Events (NCI CTCAE, version 5.0). Severe adverse events (SAEs) are defined as death, hospitalization or prolonged hospitalization, permanent or severe disability, congenital anomalies, or other significant clinical sequelae.

Exploratory biomarkers will be collected at prespecified timepoints from tumor tissue (CD8^+^ T−cell density; HIF−1α and CAIX), blood (CD4%, CD8%, and the CD4:CD8 ratio), and fecal samples (Shannon diversity index); analyses are descriptive and hypothesis−generating, as detailed in **Supplementary File 1**. Samples will be processed and stored in a secured biobank following standard operating procedures. Any future research using stored samples will require separate ethical approval.

### Study population and eligibility criteria

#### Inclusion criteria:

1. Diagnosis: Histologically or cytologically confirmed adenocarcinoma of the stomach or gastroesophageal junction (including signet ring cell carcinoma, mucinous adenocarcinoma, and hepatoid adenocarcinoma).
2. Disease status: The presence of metastatic disease, attributable to either recurrence or distant dissemination, was established through a combination of radiologic or surgical assessments.
3. Survival Expectancy: Predicted to live more than 3 months.
4. Age: 18–75 years.
5. Prior treatments:
  1. There are no previous antitumor treatments (chemotherapy, radiotherapy, targeted therapy, immunotherapy, interventional therapy, etc.).
  2. If patients previously received adjuvant or neoadjuvant therapy, the last treatment must have been completed at least 6 months before enrollment, with no recurrence or disease progression during treatment.
  3. Palliative radiotherapy is allowed if it is completed at least 2 weeks before the first study treatment.
  4. The use of prior anti-tumor traditional Chinese medicine is allowed if it is discontinued at least 2 weeks before enrollment.
6. Performance Status: ECOG PS ≤1.
7. Assessable lesion: At least one measurable lesion per the RECIST 1.1 criteria.
8. Pathological samples: Patients whose archived or fresh pathological tissue was obtained within 6 months before signing informed consent, which was sufficient for PD-L1 testing with obtainable results, were included.
9. Organ function:
  1. Hematology (no transfusion or G-CSF use within 14 days before screening):
    Hemoglobin ≥90 g/L.
    Absolute neutrophil count (ANC) ≥1.5×10^9^/L.
    Platelet count ≥75×10^9^/L.
  2. Biochemistry (no albumin use within 14 days before screening):
    Albumin ≥28 g/L.
    Total bilirubin ≤1.5×ULN.
    AST and ALT levels were ≤3×ULN (≤5×ULN if liver metastasis was present).
    Creatinine ≤1.5×ULN.
  3. Coagulation:
    INR or PT ≤1.5×ULN.
    APTT ≤1.5×ULN.
10. Systemic treatment history: No systemic treatment (including adjuvant/neoadjuvant) was given within the past 6 months after sample collection for enrollment.
11. Toxicity: Prior antitumor treatment or surgery-related acute toxic reactions were resolved to grade 0–1 per NCI CTCAE v5.0 or to levels specified by the inclusion/exclusion criteria.
12. Contraception: Strict contraception measures.
13. Consent and Compliance: Signed informed consent, willing and able to comply with study visits, treatments, lab tests, and procedures.

#### Exclusion criteria:

1. HER2 status: HER2-positive (HER2 3+ or 2+ & FISH+).
2. Tumor type: Nonadenocarcinoma gastric cancers, including squamous cell carcinoma, undifferentiated carcinoma, or mixed histological types.
3. CNS Metastasis: Uncontrolled or symptomatic active CNS metastasis (e.g., clinical symptoms, brain edema, spinal cord compression, carcinomatous meningitis, soft meningeal disease, or progressive growth).
4. Fluid accumulation: Uncontrolled pleural effusion or ascites treated with drainage within 14 days before enrollment; symptomatic or moderate to large pericardial effusion.
5. Weight loss: Weight loss >20% within 2 months before enrollment.
6. Recent treatments:
  1. Major surgery within 28 days before enrollment (diagnostic biopsies and PICC placement allowed).
  2. Immunosuppressive drugs should be used within 7 days before enrollment, excluding nasal/inhaled corticosteroids or physiological-dose systemic steroids (≤10 mg/day prednisone or equivalent).
  3. Live attenuated vaccines were administered within 28 days before enrollment, during the study, or within 60 days after treatment ended.
  4. Antitumor treatments were administered within 28 days before enrollment (chemotherapy, radiotherapy, immunotherapy, endocrine therapy, targeted therapy, biological therapy, or tumor embolization).
7. Other Malignancies: Patients were diagnosed with any other malignancy within 3 years before study entry, except for localized and cured basal cell carcinoma, squamous or superficial bladder carcinoma, cervical carcinoma *in situ*, ductal carcinoma *in situ* of the breast, and papillary thyroid carcinoma.
8. Autoimmune Diseases: Any active, known, or suspected autoimmune disease. Stable conditions not requiring systemic immunosuppression are allowed (e.g., type I diabetes; hypothyroid diabetes managed with hormone replacement; and skin diseases not needing systemic treatment, such as vitiligo, psoriasis, and alopecia).
9. Neurological/Psychiatric Conditions: Uncontrolled epilepsy, congenital spherocytosis, claustrophobia, or angle-closure glaucoma.
10. Immune therapy history: Prior treatment with anti-PD-1/PD-L1 antibodies, anti-CTLA-4 antibodies, or other T-cell costimulation/checkpoint pathway drugs.
11. Bleeding/thrombosis events: significant bleeding symptoms or tendencies within 3 months before enrollment; gastrointestinal perforation or fistula within 6 months; thrombotic events (e.g., stroke, deep vein thrombosis, pulmonary embolism) within 6 months.
12. Major Vascular Disease: Major vascular disease within 6 months before study treatment (e.g., aortic aneurysm needing surgery or recent peripheral arterial thrombosis).
13. Wounds and fractures: Severe, unhealed, or open wounds; active ulcers; or untreated fractures.
14. Neuropathy: Peripheral neuropathy >Grade 1.
15. Intestinal Obstruction: History of intestinal obstruction or related symptoms within 6 months before study treatment. Patients treated surgically to resolve incomplete obstructions at initial diagnosis may be included.
16. Systemic diseases: Interstitial lung disease, noninfectious inflammation, or uncontrolled systemic diseases (e.g., diabetes, hypertension, pulmonary fibrosis, acute pneumonia).
17. Drug Allergies: Patients with known severe allergic reactions to the study drugs or any monoclonal antibodies.
18. HIV/AIDS: HIV infection or known AIDS.
19. Hepatitis: Untreated active hepatitis B (HBV-DNA ≥500 IU/ml), hepatitis C (HCV-RNA above the detection limit), or coinfection with HBV and HCV.
20. Cardiac conditions: Myocardial infarction, severe/unstable angina, NYHA Class II or higher heart failure, significant arrhythmias, or congestive heart failure within 6 months before enrollment.
21. Hypertension: Poorly controlled hypertension despite treatment (systolic BP >140 mmHg or diastolic BP >90 mmHg).
22. Infections and Fever: Systemic antibiotic use ≥7 days within 4 weeks before enrollment or unexplained fever >38.5 °C during screening/before the first dose (tumor-related fever allowed on the basis of the investigator’s judgment).
23. Transplant history: Known history of allogeneic organ or hematopoietic stem cell transplantation.
24. Other Clinical Trials: Participation in any other drug clinical trial within 4 weeks before enrollment or within 5 half-lives of the last study drug.
25. Substance Abuse: History of psychiatric drug abuse or drug addiction.
26. Other Severe Conditions: Any other serious physical or mental illness, abnormal lab tests increasing study risk or interfering with results, or deemed unsuitable by the investigator.
27. Pulmonary conditions including a history of treated pneumothorax, severe emphysema, or pulmonary bullae.

### Intervention

**Figure 2:**
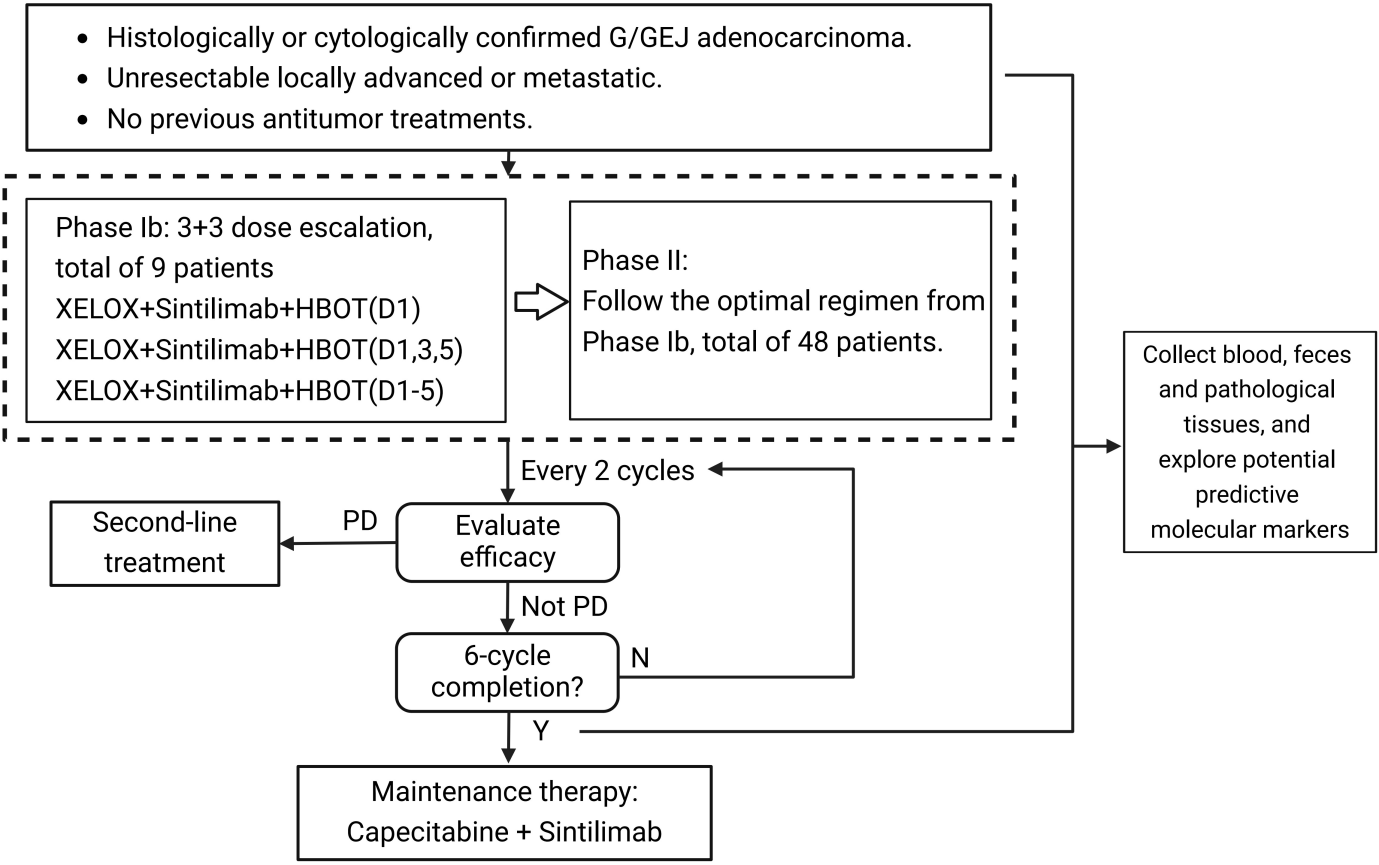
Schematic overview of the phase Ib/II trial design. Patients with pathologically confirmed, unresectable, HER2−negative advanced or metastatic gastric or gastroesophageal junction adenocarcinoma receive first−line therapy. Phase Ib uses a 3 + 3 design to evaluate HBOT schedules and select the recommended Phase II dose. Phase II enrolls 48 patients who receive six 21−day cycles of XELOX plus sintilimab and HBOT at the selected dose, with imaging every two cycles. Patients without progression after six cycles transition to maintenance with capecitabine plus sintilimab, and HBOT is not continued. Patients who progress at any time discontinue protocol therapy and proceed to subsequent treatment.

All patients who underwent imaging or pathological evaluation and, if they met the inclusion criteria, were enrolled and provided informed consent. Enrolled patients received a combined treatment regimen of XELOX chemotherapy, sintilimab immunotherapy, and HBOT. The XELOX regimen consisted of oxaliplatin 130 mg/m² administered by intravenous infusion on Day 1 and capecitabine 1000 mg/m² taken orally twice daily on Days 1–14 of each 3-week cycle. Sintilimab was given as a 200 mg intravenous infusion on Day 1 of each cycle. On Day 1 of each cycle, patients first received the sintilimab infusion followed by the oxaliplatin infusion, and immediately after completing these infusions, they underwent a 1-hour HBOT session at 2 ATA later the same day. Efficacy was assessed every two cycles. After six cycles, patients with tumor progression exited the study to receive further treatment, whereas those without progression continued maintenance therapy with capecitabine plus sintilimab until disease progression, intolerable toxicity, initiation of a new antitumor treatment, withdrawal of consent, loss to follow-up, death, or any other investigator-determined reason for discontinuation, whichever came first. If any component of the treatment was discontinued for any reason, the remaining components were continued as appropriate. If a participant becomes resectable during therapy and undergoes surgery per MDT decision, study treatments may be held or discontinued to allow surgery. The participant remains on study and transitions to the follow-up phase, with continued assessments of safety and survival endpoints. **Figure 2** illustrates the study design schematic.

Treatment adherence will be ensured by maintaining drug accountability records for capecitabine and infusion records for oxaliplatin and sintilimab. HBOT attendance will be logged at each session. Study staff will monitor adherence at every visit, and any deviations from the protocol will be recorded and evaluated. Supportive care measures, such as antiemetics, antibiotics, and analgesics, will be permitted as clinically indicated. The use of corticosteroids is allowed only at physiological doses (≤10 mg/day of prednisone or equivalent). Concomitant anticancer therapies, including radiotherapy, targeted therapy, and additional immunotherapies, are prohibited during the trial. Participants will be recruited from outpatient and inpatient services at West China Hospital through clinician referrals, review of clinic schedules, and study advertisements. Regular reminders via telephone or electronic messaging will promote participant retention. For participants who discontinue the intervention prematurely, available outcome data will be collected up to the point of withdrawal for analysis.

### Safety monitoring

Safety monitoring for all treatments in this study, including chemotherapy, immunotherapy, and HBOT, is conducted in accordance with CTCAE v5.0 criteria. In particular, for HBOT-related toxicity, predefined criteria for early treatment interruption or permanent discontinuation, as well as procedures for monitoring and managing severe adverse events, have been established. The plan also defines SAE identification, documentation, expedited reporting, and escalation pathways. Full procedures and dose−modification tables (including Chemotherapy, Immunotherapy, and HBOT) are provided in **Supplementary File 2**. Investigators will evaluate, document, and report all AEs in real time, follow patients to resolution/stability, and act according to protocol−defined thresholds.

### Statistical analysis

#### Analysis populations

Three analysis populations were defined for this study: the Intent-to-Treat (ITT) population, the Per-Protocol (PP) population, and the Safety population. The ITT population includes all patients who provided informed consent and received any amount of study treatment. In practice, this means patients who received at least one dose of the study therapy or underwent at least one HBOT session. The PP population consists of patients who met all key eligibility criteria, had no major protocol violations, completed at least two treatment cycles, and had evaluable data for the primary endpoint. The Safety population comprises all patients who initiated study treatment, meaning they received at least one dose of the study drug or at least one HBOT session.

Efficacy analyses will primarily use the ITT population, while the PP population will be analyzed in a sensitivity context to assess the impact of protocol deviations. All safety analyses will use the Safety population. In addition, patients from the Phase Ib portion of the trial who received the same HBOT regimen as selected for Phase II will be combined with the Phase II patients to form a pooled RP2D analysis set. This combined dataset will be used for supportive analyses of efficacy and safety endpoints. The pooled Phase Ib+II results will be presented alongside the Phase II–only results to provide additional context, but they will not serve as the primary basis for any efficacy conclusions and will not influence the primary analysis in the Phase II ITT population. Tables of results will clearly indicate whether each patient came from Phase Ib or Phase II.

#### Baseline characteristics

Patient demographics and baseline disease characteristics will be summarized using descriptive statistics. These characteristics include age, sex, tumor location, histological subtype and tumor differentiation grade, TNM stage, baseline tumor burden, PD-L1 expression status, mismatch repair (MMR) status, and prior treatments. Continuous variables will be reported as the mean and standard deviation (SD) if the data are approximately normally distributed, or as the median and interquartile range (IQR) if the distribution is skewed. Categorical variables will be summarized as counts and percentages.

#### Efficacy endpoints

This is a single-arm exploratory trial; therefore, all efficacy results will be descriptive with no formal between-group comparisons. The primary efficacy endpoint for Phase II is the ORR, defined as the proportion of patients achieving a best overall response of CR or PR as determined by investigator assessment according to RECIST v1.1. The first tumor assessment is scheduled at approximately 6 weeks of therapy, which corresponds to the end of two treatment cycles. ORR will be reported along with a two-sided 95% exact confidence interval calculated using the Clopper–Pearson method. In the ITT analysis, patients with no post-baseline tumor assessments will be considered non-responders for the purpose of calculating ORR. The observed ORR will be compared qualitatively to historical outcomes from first-line therapy for advanced GC/GEJC; however, no formal hypothesis testing will be performed due to the single-arm study design.

Secondary efficacy endpoints will be analyzed and reported descriptively. PFS is defined as the time from treatment initiation until radiologic disease progression or death. It will be estimated using the Kaplan-Meier method; the median PFS and its two-sided 95% confidence interval will be reported. Patients who are alive without progression at the time of analysis will be censored at the date of their last disease assessment. DCR is the percentage of patients achieving CR or PR or sustained stable disease for at least 6 weeks. DCR will be reported with a two-sided 95% exact confidence interval calculated by the Clopper-Pearson method. For the subset of patients who undergo curative-intent surgery (R0 resection) after successful therapy, a 2-year DFS rate will be calculated. This rate will be defined as the proportion of those post-surgery patients who are alive and recurrence-free 24 months after surgery. Because this subset is expected to be small, the 2-year DFS will be presented as a descriptive metric without formal statistical comparison. OS is defined as the time from treatment initiation until death from any cause. The 24-month OS rate will be estimated using Kaplan-Meier methods, and if sufficient follow-up is available, the median OS and its 95% confidence interval will also be reported. If the median OS is not reached by the analysis time, the survival rate at the last follow-up time point will be provided instead. Patients who are alive at the time of analysis (or last known follow-up) will be censored on that date for OS and DFS analyses. Patient-reported quality of life outcomes will be summarized at baseline and at specified time points during treatment. Changes from baseline in QoL scores may be explored with paired statistical tests, depending on data distribution, as an exploratory analysis.

#### Safety analysis

All patients who receive any study treatment will be included in the safety analysis. AEs will be recorded at each visit and graded according to the NCI-CTCAE version 5.0. The incidence of all AEs, SAEs, and treatment-related AEs will be summarized in frequency tables by system organ class and preferred term, with the number and percentage of patients affected in each category. Key adverse event rates, such as the incidence of Grade 3 or higher toxicities, will be reported with corresponding 95% confidence intervals. Events that lead to dose reductions or permanent treatment discontinuation will be tabulated or listed separately. Particular attention will be given to events of special interest in this combination regimen, including immune-related AEs and any toxicities attributable to HBOT. In the absence of a control arm, the safety data will be interpreted descriptively and may be compared qualitatively to historical safety data from similar treatment regimens.

Clinical laboratory test results and vital signs will be monitored for changes of clinical significance over time. Continuous laboratory parameters will be analyzed for changes from baseline using paired statistical tests: if a parameter is approximately normally distributed, a paired t-test will be used; if not, the Wilcoxon signed-rank test will be used as a non-parametric alternative. Summary statistics (mean ± SD or median with IQR) for laboratory values may be presented at baseline and at post-baseline time points, along with the change from baseline. Categorical shifts in laboratory values (such as the proportion of patients whose results shift from normal to abnormal ranges) will be summarized as counts and percentages in contingency tables.

##### Exploratory Biomarker Analysis

Biomarker samples will be collected at prespecified time points from tumor tissue, peripheral blood, and fecal material (see **Supplementary File 1** for detailed collection and analysis procedures). Key exploratory biomarkers include tumor tissue parameters (intratumoral CD8^+^T-cell density and expression of hypoxia-related markers HIF-1α and CAIX), peripheral blood immune cell subsets (CD4^+^ and CD8^+^ T-cell percentages and the CD4:CD8 ratio), and baseline gut microbiome diversity indices (the Shannon diversity index from fecal samples). All biomarker analyses will be descriptive and hypothesis-generating. Analyses will be performed on the subset of patients who have evaluable data for both the biomarker and clinical outcome of interest; for each biomarker endpoint, the number of patients with available data (n/N) will be reported. We will explore changes in biomarker levels over time and their associations with clinical outcomes, but these exploratory findings will be interpreted with caution. No formal hypothesis testing is planned for biomarker endpoints in the main analysis.

#### Subgroup analyses

No inferential subgroup comparisons or covariate-adjusted analyses are planned due to the limited sample size. However, to aid clinical interpretation, key efficacy outcomes will be presented descriptively across several predefined patient subgroups (display-only analyses for exploratory purposes). Subgroups of interest include: (i) tumor location (gastric vs gastroesophageal junction), (ii) presence of baseline liver metastases (yes vs no), (iii) PD-L1 combined positive score (CPS ≥1 vs <1, with an additional exploratory cut-off of CPS ≥5 vs <5 if sample size permits), and (iv) tumor MMR status (pMMR vs dMMR). Within each subgroup, ORR will be reported as n/N (%) with two-sided 95% exact confidence intervals (Clopper–Pearson method). The absolute difference in response rates between subgroup categories and the corresponding odds ratio will also be provided as point estimates without calculation of p-values or formal statistical tests. PFS in each subgroup will be illustrated using Kaplan-Meier curves and summarized by the median PFS (with 95% CI). Patients missing the relevant subgroup or biomarker information will be excluded from that subgroup analysis, and no data imputation will be performed for missing subgroup classification variables. All subgroup analyses are considered exploratory and descriptive; no significance tests or interaction tests will be conducted, and these subgroup results will not affect the pre-specified primary and secondary endpoint analyses.

#### Sensitivity analyses and missing data

##### Sensitivity analyses

The robustness of the primary endpoint analysis will be examined through predefined sensitivity analyses. In addition to the main ORR analysis conducted on the ITT population, the ORR will also be calculated for the PP population to evaluate the impact of any protocol deviations on the primary outcome. Furthermore, as an exploratory sensitivity analysis concerning PD-L1 status, the effect of using an alternative PD-L1 expression cutoff will be assessed: specifically, outcomes will be examined using a CPS threshold of 5 (CPS ≥5 vs. <5), as was prespecified before the trial.

##### Missing data

For the primary endpoint ORR in the ITT population, patients who have no post-baseline tumor assessments will be counted as non-responders. For time-to-event endpoints such as PFS, DFS, or OS, patients who have not experienced the event by the cutoff date will be censored at the time of their last adequate disease assessment. If scheduled visits or assessments are missed, all data collected up to the point of patient withdrawal or loss to follow-up will be included in the analysis, and no forward imputation of missing future observations will be performed. Missing data in patient-reported outcome measures, such as QoL questionnaires, will not be imputed; analyses will be based on available data only.

To address the potential impact of missing data, we may conduct additional exploratory analyses using methods that accommodate data missing at random. For example, a linear mixed-effects model for repeated measures could be employed to explore longitudinal trends in QoL scores despite some missing follow-up data. If more than 10% of patients have missing data for the primary endpoint, we will perform additional sensitivity analyses to ensure the robustness of our conclusions. These may include using multiple imputation techniques to estimate missing outcomes and conducting best-case and worst-case scenario analyses to gauge the potential range of impact due to the missing data.

#### Phase Ib analysis

In the Phase Ib safety run-in portion, no formal statistical hypothesis testing will be performed due to the small sample size. Selection of the recommended Phase II dose (RP2D) HBOT regimen will be guided primarily by safety and tolerability outcomes (the occurrence of any DLTs during the first treatment cycle, the incidence of ≥Grade 3 HBOT-related adverse events, and the frequency of treatment interruptions or dose reductions attributable to toxicity). Feasibility metrics, such as patient adherence to the HBOT schedule (compliance rate and completion of planned sessions), and preliminary efficacy signals (ORR and DCR, assessed descriptively) will also be taken into account in determining the RP2D. If multiple HBOT regimens exhibit similar safety profiles, the regimen with better feasibility and a more favorable preliminary efficacy trend will be selected for progression to the Phase II trial.

### Recruitment strategies

Participants will be recruited from the outpatient and inpatient services of the West China Hospital, Sichuan University. Eligible patients will be identified through referral by treating oncologists, review of clinic schedules, and pre-screening of electronic medical records.

## Discussion

The treatment landscape for advanced GC/GEJC has entered the era of immunotherapy. Compared with chemotherapy alone, combining ICIs with chemotherapy has been shown to prolong OS and PFS as a first-line treatment for advanced or metastatic GC/GEJC. This combination is recommended as a standard treatment in most guidelines (3–5, 25). However, response rates to ICI and chemotherapy combinations remain modest, and many patients who initially respond quickly develop resistance, resulting in overall efficacy that falls short of clinical expectations. Therefore, there is an urgent need for new combination treatment strategies. Although various ICI-sensitizing combination therapies are in different stages of clinical research, existing data suggest that these strategies often lead to higher rates of adverse reactions (26, 27). In contrast, HBOT can improve the hypoxic tumor microenvironment by enhancing drug delivery to the tumor, promoting the infiltration of CTLs, and reversing the immunosuppressive environment, thereby increasing antitumor responses (11–13). Additionally, compared with other combination therapies, HBOT has fewer contraindications, lower rates of adverse reactions, and a lower economic burden, making it a highly promising treatment strategy.

We reviewed clinical studies on the use of HBOT in cancer treatment. Although evidence suggests that HBOT can enhance the efficacy of radiotherapy and chemotherapy, studies are limited and often involve small sample sizes, with HBOT primarily used as an adjunct to these therapies. For example, Hongmei Xing reported that, compared with control patients, postoperative glioma patients receiving HBOT combined with chemotherapy presented significantly improved motor function (FMA) and activities of daily living (ADL) scores. In radiotherapy, HBOT has been shown to significantly reduce radiation-induced proctitis and cystitis and prevent radiation-induced osteonecrosis (28, 29). These studies indicate that HBOT combination therapies may not increase adverse reactions and may even improve patients’ quality of life. In our trial, including quality-of-life as a secondary endpoint allows us to directly evaluate this aspect. If improved QoL is observed alongside tumor response, it will underscore the patient-centered benefits of adding HBOT. This information can guide clinicians in weighing the regimen’s value beyond traditional efficacy metrics.

In our previous studies of advanced GC/GEJC, we observed that a subset of patients became eligible for surgery after systemic therapy. To further evaluate long−term benefit in this patient cohort, we will prospectively follow patients who undergo surgery and include two−year DFS as a secondary endpoint restricted to those converted to resectable disease and achieving an R0 resection. This endpoint is intended to assess the durability of disease control and the potential curative benefit of conversion therapy.

Currently, there is no consensus on the optimal pressure and oxygen duration for the use of HBOT in tumor treatment. Our review of multiple clinical and preclinical studies revealed that most used pressures between 2.0–2.5 ATA and oxygen inhalation times of 30–90 minutes (29–33). For gastrointestinal tumors, Qi Sun et al. administered HBOT at 2.0 ATA for 60 minutes, and the results demonstrated good safety and a lower incidence of adverse reactions than in controls. Owing to the significant variability in HBOT treatment protocols, our study employs a 3 + 3 dose-escalation design and a single-arm exploratory approach to assess the safety, tolerability, and preliminary efficacy of HBOT combined with chemotherapy and ICIs in the early stages. This phased assessment will help optimize treatment strategies and provide a solid data foundation for future randomized controlled trials.

As a key intervention in this study, HBOT may offer substantial antitumor benefits but can also cause potential adverse reactions, such as pneumothorax, ear barotrauma, chest tightness, headaches, and blurred vision (34). Therefore, strict monitoring of patients during treatment is essential, particularly for respiratory status and ear responses. Early identification of adverse reactions, rational treatment design, and timely interventions can effectively minimize HBOT risks and ensure patient safety. Additionally, the potential for organ-specific adverse events in GC/GEJC patients receiving HBOT is unknown, necessitating close monitoring throughout treatment.

However, this study has several limitations. It is designed as a single-arm, single-center clinical trial without a control group, which limits the ability to directly attribute observed clinical outcomes to the addition of HBOT. The relatively small sample size may restrict the statistical power and the generalizability of the findings to broader populations. Furthermore, the open-label nature of the trial and potential biases related to patient selection and center-specific clinical practices cannot be completely excluded. These limitations should be considered when interpreting the results and planning future confirmatory studies.

In summary, we are conducting a prospective, single-center, single-arm phase Ib/II clinical trial to evaluate the efficacy and safety of HBOT combined with chemotherapy and immunotherapy in HER2-negative advanced or metastatic GC/GEJC. To our knowledge, no clinical studies have reported the use of HBOT combined with ICIs for tumor treatment, suggesting a novel therapeutic strategy for patients with advanced GC/GEJC.
