# Supplementary material for: XELOX combined with sintilimab and hyperbaric oxygen therapy for advanced or metastatic gastric/gastroesophageal junction adenocarcinoma: study protocol for a prospective, single-arm, phase Ib/II clinical trial
Source: Front Immunol. 2026 Jan 12;16:1672725. doi: 10.3389/fimmu.2025.1672725 (PMC12833279; doi:10.3389/fimmu.2025.1672725)
Supplement: Supplementary file 1 [file DataSheet1.pdf]

Exploratory Biomarker Collection and Analysis Plan

1. Sample Types and Collection Timepoints

In this study, three types of biological samples (tumor tissue, peripheral blood, and feces) will be collected at scheduled timepoints for exploratory biomarker analysis. Specifically:

1.1 Tumor Tissue Samples (FFPE)

- **T0 (Baseline):** Within 0-7 days before the start of treatment (preferably within 0-3 days), obtain a formalin-fixed paraffin-embedded (FFPE) tumor tissue sample via gastroscopic biopsy.
- **T1 (After 1 cycle):** Within 0-7 days before Cycle 2 Day 1 (C2D1) of treatment (preferably within 0-3 days), repeat the gastroscopic biopsy to obtain an FFPE tumor sample.
- **At PD (Disease Progression):** An optional FFPE tumor biopsy at the time of disease progression, if the subject consents (not mandatory).

Note: Collect 4-6 tumor tissue pieces from the same lesion whenever possible. Record the tumor cell content (≥20% recommended) and the sampling site to facilitate subsequent quantitative analyses.

1.2 Peripheral Blood Samples

- **T0 (Baseline):** Within 0-7 days before the start of treatment (preferably on Day 1 of Cycle 1), collect 5-10 mL of blood in an EDTA tube. Concurrently perform a complete blood count (CBC) on the same day to allow conversion of subset percentages to absolute counts.
- **T1 (After 1 cycle):** Within 0-7 days before C2D1 (preferably on C2D1), collect another 5-10 mL EDTA blood sample using the same method.
- **At PD (Disease Progression):** An optional peripheral blood draw at disease progression, if the subject consents (not mandatory).

Note: Isolate peripheral blood mononuclear cells (PBMCs) within ≤2 hours after blood draw, and perform flow cytometry on the same day if possible. If immediate analysis is not feasible, cryopreserve the PBMCs in liquid nitrogen for batch analysis at a later time. All blood samples should be drawn prior to the day's drug administration, infusion, or hyperbaric oxygen therapy (HBOT) to minimize short-term fluctuations from these interventions.

1.3 Fecal Samples

- **T0 (Baseline):** Collect one fresh stool sample within 0-7 days before the start of treatment. Instruct the subject to use a sterile container to collect a fresh fecal sample, avoiding urine contamination. Store the sample at 4°C and transfer it to -80°C within 24 hours of collection.

Note: Document potential confounding factors for stool samples, including any antibiotic, proton pump inhibitor (PPI), or probiotic use in the past 8 weeks, as well as any recent acute gastrointestinal infections.

2. Planned Biomarker Analyses

In line with the sample types above, this study plans to analyze a variety of exploratory biomarkers to evaluate the tumor immune microenvironment, the systemic immune status, and the gut microbiota characteristics of the patients. The specific biomarkers to be assessed for each sample type are as follows:

2.1 Tumor Tissue

Markers will be evaluated to characterize the extent of immune cell infiltration and hypoxic status in the tumor microenvironment. Specifically, we will measure immune infiltration-related markers (such as tumor-

infiltrating lymphocyte levels, including total $CD3^+$ T cells, the infiltration density of $CD8^+$ cytotoxic T cells, the number of $CD68^+$ tumor-associated macrophages, etc.) as well as tumor hypoxia-related markers (such as the expression levels of hypoxia-inducible factor-1α (HIF-1α) and carbonic anhydrase IX (CAIX)). These biomarkers will help delineate whether the tumor immune microenvironment is in an active or suppressive state.

Among the many candidate indicators, this study will **focus on** the $CD8^+$ T cell infiltration density in tumor tissue and the expression of HIF-1α and CAIX (which reflect the degree of tumor hypoxia), as these are thought to be most closely associated with treatment response. Other immune-infiltration markers (such as total $CD3^+$ T cell count, $CD68^+$ macrophage count, etc.) will also be measured, but primarily as background reference information. They will not serve as main variables in the efficacy endpoint analyses.

2.2 Peripheral Blood

Biomarkers will be analyzed to characterize the composition of immune cell subsets in peripheral blood and related markers, reflecting the patient's systemic immune status. The analysis will mainly determine the relative proportions and absolute counts of various immune cell subsets, including T lymphocytes ($CD3^+$ total T cells, $CD4^+$ helper T cells, $CD8^+$ cytotoxic T cells), B lymphocytes ($CD19^+$ B cells), natural killer (NK) cells ($CD16^+$/$CD56^+$ NK cells), monocytes/myeloid cells ($CD14^+$ monocytes, myeloid-derived suppressor cells [MDSCs]), and other key subsets. In particular, the study will focus on the distribution and balance of T cell subsets, for example, the percentage of $CD8^+$ T cells, the percentage of $CD4^+$ T cells, and the CD4/CD8 ratio, since these measures have been suggested in previous research to influence anti-tumor immune responses and patient prognosis. Other cell subset indices (such as B cell and NK cell levels) will also be measured but will serve primarily as background information and will not be included as main variables in the primary endpoint analyses.

2.3 Fecal Samples

Biomarkers will be analyzed to characterize the composition and diversity of the gut microbiota, in order to evaluate potential relationships between the gut microbiome and treatment efficacy. The planned metrics include measures of gut microbiota α-diversity (the Shannon diversity index, reflecting the diversity of an individual's microbiome) and microbiota compositional characteristics (the relative abundance distribution of major microbial phyla and genera). This study will pay special attention to certain bacterial genera that have been frequently reported in the context of cancer immunotherapy and are potentially associated with treatment outcomes - in particular, probiotic genera such as *Akkermansia* and *Bacteroides*. In addition, the overall baseline diversity of the fecal microbiota is of interest (with the hypothesis that higher microbiome diversity may indicate a more favorable immune milieu). The above key indices (diversity measures and the abundance of specific genera) will be prioritized for statistical correlation analyses. Other microbiome metrics (such as the abundances of other genera) will also be documented and described, but only as background reference and not as primary variables in statistical analyses.

3. Detection Methods and Experimental Workflow Overview

For each type of sample and its associated biomarkers, multiple laboratory techniques will be employed. The main methods and workflows are summarized below:

3.1 Tumor Tissue Analysis

For FFPE-fixed tumor tissue sections (approximately 4 μm thick), a combination of multiplex immunofluorescence (mIF) and immunohistochemistry (IHC) will be used to detect the target biomarkers. First, a multiplex immunofluorescence technique is applied to simultaneously stain and evaluate multiple immune cell and protein markers on the same slide. Using an automated staining platform, specific primary antibodies against T cells, B cells, macrophages, etc., along with corresponding fluorescent-labeled secondary antibodies (including DAPI for nuclear counterstaining), are applied to each slide, enabling the detection of up to 5-6 different protein markers in one run. After staining, the slide is scanned with a whole-slide fluorescence

imaging system, and image analysis software is used to quantitatively calculate the number of positive cells per unit area and the fluorescence signal intensity, thereby quantifying parameters such as $CD8^+$ T cell infiltration density. In this analysis, digital pathology methods are employed. For example, to assess $CD8^+$ T cell infiltration, standardized regions of interest (ROIs) are defined in both the tumor core and the invasive margin, and the result is reported in cells/mm².

For certain markers like HIF-1α and CAIX, separate IHC assays will be performed (on individual tissue sections) to determine their expression. The IHC-stained slides are evaluated using a semi-quantitative H-score (0-300). Each IHC slide is independently reviewed by two trained pathologists who score the staining according to predefined criteria; if their scores differ by more than 15%, the slide is re-examined jointly under a microscope and discussed to reach a consensus. By combining the mIF and IHC approaches described above, the study ensures a reliable assessment of multiple exploratory biomarkers in the tumor tissue.

3.2 Peripheral Blood Analysis

Peripheral blood samples will be processed in the laboratory as soon as possible after collection (within ≤2 hours). First, peripheral blood mononuclear cells (PBMCs) are isolated by density gradient centrifugation (using Ficoll-Paque). The isolated PBMCs can be used immediately for multi-parameter flow cytometry analysis or cryopreserved in liquid nitrogen for later batch analysis. For flow cytometry, an aliquot of cells from each sample is incubated with a cocktail of fluorescently labeled antibodies that mark different types of immune cell subsets. The staining antibody panel includes specific markers for T cells (CD3, CD4, CD8), B cells (CD19), NK cells (CD16/CD56), and monocytes/granulocytes or other myeloid cells (CD14, CD15). After staining, data are acquired on a flow cytometer, capturing multi-color fluorescence parameters for each cell.

Subsequently, the flow cytometry data are analyzed using FlowJo software according to a pre-established gating strategy. The analysis workflow sequentially excludes cell debris and dead cells, then gates on the lymphocyte population, and thereafter identifies the T cell, B cell, and NK cell populations, as well as further subdivisions of subsets (for example, within the $CD4^+$ and $CD8^+$ T cell populations, distinguishing effector and memory subsets). For each sample, at least 1×10^5 cell events are collected to ensure the reliability of the statistics. The final output yields the relative proportion of each immune cell subset as a percentage of total PBMCs, as well as the corresponding absolute count for each subset (calculated by referencing the CBC results). These immune profiling data will be used for subsequent statistical analyses.

3.3 Fecal Microbiota Analysis

For the frozen fecal samples, 16S rRNA gene sequencing of the gut microbiota will be performed in batches. First, total DNA is extracted from the stool using a commercial kit according to standard procedures (the protocol includes mechanical homogenization and multiple purification steps to improve microbial DNA yield). The hypervariable V3-V4 region of the bacterial 16S rRNA gene is then targeted for PCR amplification. After purifying the PCR products, sequencing libraries are constructed and subjected to paired-end sequencing on a high-throughput platform (Illumina MiSeq), generating 16S rRNA sequence data for each stool sample.

Next, a bioinformatics pipeline is applied to the sequencing data for quality control and taxonomic annotation. Specific steps include: performing quality filtering on the raw reads and merging paired-end reads, and removing chimeric sequences; then clustering the high-quality sequences into operational taxonomic units (OTUs) or binning them as amplicon sequence variants (ASVs); and comparing these sequences against a 16S reference database to annotate each OTU/ASV with its taxonomic classification (kingdom, phylum, class, order, family, genus, species). Based on the taxonomic annotations, the relative abundance of each taxon in each sample is calculated. Diversity indices are then computed, including each sample's α-diversity (Shannon diversity index) and the β-diversity between samples (to compare differences in microbiota composition between individuals). Throughout the sequencing process, negative controls are included at the PCR amplification and library preparation steps to monitor for environmental contamination, thereby ensuring the reliability of the final gut microbiota profiles.

4. Sample Handling and Quality Control Measures

To ensure the reliability and validity of experimental results, stringent quality control (QC) measures are implemented during sample handling and testing:

4.1 Tumor Tissue Sample QC

All tumor tissue samples, upon being sent to the lab, are first subjected to quality assessment by the pathology department. The focus is on confirming that the tissues were properly fixed and preserved (e.g., appropriate duration of formalin fixation, paraffin blocks free of moisture damage or cracks), and selecting a paraffin block with sufficient tumor cell content for sectioning. Multiple consecutive sections are cut for each sample; one section is H&E stained and reviewed by a pathologist to confirm the tumor area and tumor cell content (with a requirement that tumor cell content ≥20% to ensure enough tumor cells for evaluating immune infiltration). The remaining sections are used for subsequent mIF/IHC staining.

During immunostaining, each batch of slides includes positive and negative control slides: the positive control is a tissue sample known to highly express the target antigen, and the negative control is processed with the primary antibody omitted to check for nonspecific background staining. After staining is completed, prior to image analysis, the scanned images are inspected to ensure they are well-focused, that the fluorescence or chromogenic signals are normal, and that there is no obvious nonspecific background staining. For the critical IHC results of HIF-1α and CAIX, two independent observers (blinded to each other's results) review the slides and record their scores to improve interpretative consistency. If the two scores differ by more than a preset threshold (>15% difference in H-score), the observers will jointly re-examine the slide under a microscope and discuss to reach an agreement. Additionally, if any staining intensity score or positive cell count for a tissue sample appears abnormal, the experiment (or slide preparation) will be repeated immediately for that sample to maximize data accuracy and reliability.

4.2 Peripheral Blood Sample QC

Peripheral blood samples are collected following standard operating procedures, and PBMC isolation is completed as quickly as possible (within 2 hours) to reduce cell apoptosis and functional alterations. During the isolation process, room temperature conditions and sterile technique are maintained. The total yield and viability of the isolated cells are assessed by trypan blue exclusion (with a cell viability of ≥90% generally required to proceed to downstream analyses). Before running each flow cytometry session, standard calibration beads are used to calibrate the cytometer's lasers and detectors, ensuring consistency of signal detection over time. For multicolor flow cytometry, single-stained controls and compensation controls are used to set accurate compensation parameters. Each sample is stained using the same antibody concentrations and incubation times, and after staining, the cell suspension is passed through a filter to remove any clumps, thus avoiding clogging of the flow cytometer. During data analysis, a gating strategy is pre-determined using representative samples and then applied uniformly to all samples. For certain subsets where the gating is not clear-cut, two analysts independently gate the same data file and compare results to verify consistency. If a particular cell subset proportion for a sample is found to be markedly outside of expected ranges, the raw data for that sample will be reviewed and, if necessary, the sample will be re-stained and run again to rule out technical errors. Data recording and storage follow a dual-backup principle: after each analysis, the raw FCS data files and the analysis result files are immediately backed up in two separate locations to ensure data traceability and secure preservation.

4.3 Fecal Sample QC

When a stool sample is provided by a participant, the research staff first verify the sample container's label and the recorded collection time to ensure the sample identity and timing are correct. They also note the time interval from sample collection to freezing (generally required to be ≤2 hours). Upon laboratory receipt, the sample is processed uniformly according to the predefined standard procedure.

During DNA extraction, a blank extraction tube (no sample added) is included with each batch as a negative control to monitor any background contamination introduced by reagents. At the same time, a commercial microbiome standard (containing a mixture of microbial DNA with a known composition) is periodically co-processed and sequenced to verify the accuracy of the entire extraction and sequencing workflow. High-fidelity DNA polymerase is used and strict aseptic technique is followed during PCR amplification and library construction to avoid amplification bias and external contamination. Additionally, each PCR run includes a no-template negative control well to monitor for any nonspecific amplification products. For the sequencing data, a standard bioinformatics QC pipeline is employed: low-quality reads (those containing adapter sequences or with an average quality score <Q20) are filtered out, and any sequences that appear in the negative control samples are removed to control background noise. To avoid bias due to differences in sequencing depth, we predefine that each sample should have at least 50,000 reads; any sample with sequencing depth below this threshold will be flagged or excluded from analysis. Finally, the primary microbiome analysis results are validated by two independent analysts using different software pipelines (QIIME2 and Mothur) in parallel, with emphasis on comparing key metrics such as diversity indices and major genus abundances. Only when the conclusions from both analysis pipelines are consistent will the results for that sample be included in the final report, thereby ensuring the robustness and reliability of the gut microbiota analysis.

5. Statistical Analysis

This study will conduct exploratory analyses using **objective response rate (ORR)** as the primary efficacy endpoint. ORR is defined as the proportion of patients achieving an objective response (complete response or partial response) at approximately 6 weeks after treatment initiation (after 2 cycles), as determined by the investigators according to RECIST 1.1 criteria. Given the hypothesis-generating nature of the study, the statistical analyses will focus on descriptive results to explore potential associations between biomarkers and ORR, rather than formal inferential testing.

5.1 Biomarker and ORR Univariate Analysis

All pre-specified exploratory biomarkers will be evaluated individually for their association with ORR using univariate logistic regression models (with ORR as the binary outcome). Each biomarker will be included as a continuous independent variable in a separate logistic model to estimate its effect on achieving an objective response. The biomarkers to be analyzed include the following: the $CD8^+$ T cell infiltration density in tumor tissue, the expression levels of HIF-1α and CAIX in tumor tissue; the percentage of $CD4^+$ T cells, percentage of $CD8^+$ T cells, and the CD4/CD8 ratio in peripheral blood; and the Shannon diversity index of the fecal microbiota, the relative abundance of *Akkermansia*, and the relative abundance of *Bacteroides* in stool samples. Each biomarker will be modeled independently to calculate its association with ORR, and the results will be reported as odds ratios (ORs) with their 95% confidence intervals.

5.2 Variable Definition and Derivation

For the biomarker variables, the following definitions will be used or derived for analysis: Tumor tissue biomarkers will include both the baseline value (T0) and the change from baseline (Δ, defined as T1 minus T0) for each of the following, $CD8^+$ T cell infiltration density (cells/mm^2), HIF-1α expression (H-score), and CAIX expression (H-score). Peripheral blood biomarkers will include values at T0 and T1 for $CD4^+$ T cell percentage, $CD8^+$ T cell percentage, and the CD4/CD8 ratio (if necessary, corresponding absolute cell counts will be presented as supplementary information). Fecal microbiome biomarkers will use the baseline (T0) values, including the Shannon diversity index and the relative abundances of *Akkermansia* and *Bacteroides*. All continuous biomarker variables will be standardized as z-scores before being entered into regression models. For variables that show a highly skewed distribution, a rank transformation may be applied as a sensitivity analysis to ensure robustness.

5.3 Variable Standardization and Effect Size Representation

To facilitate direct comparison of effect sizes between different biomarkers, all continuous biomarker variables will be standardized (normalized to z-scores) in the regression analyses. In practice, this means each biomarker value will be transformed based on the overall mean and standard deviation for that variable, such that the resulting OR corresponds to the change in odds of ORR associated with a **one standard deviation increase** in the biomarker. The regression results will thus be presented as ORs (and 95% CIs) per 1 SD increase of the biomarker.

5.4 Multivariable Model and Hypothesis Testing

No multivariable model analyses will be performed in this exploratory study, and no formal hypothesis testing will be conducted. We will not include multiple biomarkers simultaneously in a single model to perform adjusted comparisons, nor will we establish any significance threshold based on p-values to judge the strength of associations (and no adjustments for multiple comparisons will be made). Likewise, no pre-specified subgroup analyses or analyses of interaction between biomarkers and treatment effect are planned, in order to avoid overpartitioning the data and potentially generating spurious findings given the limited sample size. The focus will instead be on the effect size estimates (the magnitude of the ORs and the width of their confidence intervals) to describe possible association trends between each biomarker and ORR. All findings will be interpreted as exploratory and hypothesis-generating, with the clear understanding that they require validation in future studies with larger sample sizes.

5.5 Missing Data Handling

This study will not perform imputation for missing biomarker data. If some patients lack data for a particular biomarker (due to sample quality issues or other reasons), those patients will simply be excluded from the analysis of that specific biomarker, and the analysis will be based on the patients with available data for that variable. Given the exploratory nature of the analysis and the relatively limited sample size, we chose to minimize assumptions and manipulations of the data to avoid introducing bias. Therefore, all results will be calculated from observed values only, without any imputation for missing values.

5.6 Sensitivity Analysis

Based on the distribution characteristics of certain biomarkers or relevant clinical considerations, sensitivity analyses will be conducted for some biomarkers. For example, patients may be divided into high vs. low groups according to the median value of a biomarker at baseline, and the ORR between these groups can be compared using the same univariate logistic regression framework described above. Alternatively, patients can be stratified using clinically reported threshold values for a biomarker (from literature) and then analyzed. These sensitivity analyses are intended to verify the consistency of the observed associations under different categorization methods and to assess the robustness of the findings.

5.7 Special Situations

If the sample size is very small or if a situation of complete separation occurs (where conventional logistic regression fails to provide reliable estimates), alternative methods will be employed to obtain more stable estimates. In particular, a Firth-corrected logistic regression (penalized maximum likelihood estimation) or an exact logistic regression approach will be used to re-analyze the data in such extreme cases. These methods can yield more stable OR estimates and confidence intervals under challenging conditions, making the analytical conclusions more reliable. All statistical analyses will be performed on the **biomarker-evaluable population** (patients who have both an ORR assessment and a valid measurement for the given biomarker), and each model will report the actual number of patients included in that analysis (n/N).
