# Supplementary material for: XELOX combined with sintilimab and hyperbaric oxygen therapy for advanced or metastatic gastric/gastroesophageal junction adenocarcinoma: study protocol for a prospective, single-arm, phase Ib/II clinical trial
Source: Front Immunol. 2026 Jan 12;16:1672725. doi: 10.3389/fimmu.2025.1672725 (PMC12833279; doi:10.3389/fimmu.2025.1672725)
Supplement: Supplementary file 2 [file DataSheet2.pdf]

Possible Adverse Reactions and Emergency Plan

This study will strictly follow Good Clinical Practice (GCP) guidelines for monitoring and reporting adverse events (AEs). An adverse event is defined as any unfavorable medical occurrence that arises from the time a subject signs the informed consent and enters the trial until the end of the final follow-up period, regardless of whether it is causally related to the investigational drug. This includes abnormal laboratory findings or clinically observed asymptomatic changes that are clinically significant, which are also considered AEs. Investigators should instruct subjects to proactively report any discomfort or symptoms during the study and record them in detail.

1. Recording of Adverse Events

During the trial, all AEs must be recorded in a timely, accurate, and complete manner in the case report form (CRF). The record should include: the name of the AE (or diagnosis), date and time of onset, severity, duration, date of alleviation or resolution, measures taken (e.g. whether treatment was given, whether the study drug dose was adjusted or paused), outcome (recovered, improved, not recovered, sequelae, death, etc.), and the investigator's assessment of its causal relationship to the study drug. If the same subject experiences the same type of AE multiple times, each occurrence should be recorded with its occurrence time and management. All AEs should be followed until they resolve or stabilize, and the final outcome should be documented.

2. Expected Adverse Events

Based on knowledge of the investigational treatments and prior clinical data, the investigators anticipate some common adverse events and will monitor these closely:

- **XELOX Chemotherapy (capecitabine + oxaliplatin):** Expected adverse reactions include bone marrow suppression (e.g. neutropenia, anemia, thrombocytopenia), gastrointestinal reactions (nausea, vomiting, diarrhea, oral mucositis), peripheral neurotoxicity (mainly caused by oxaliplatin, presenting as paresthesia, numbness, etc.), hand–foot syndrome (caused by capecitabine), and abnormal liver function, among others.
- **Sintilimab (anti–PD-1 immunotherapy):** Common adverse reactions include dermatologic and mucosal toxicity (rash, pruritus, etc.), gastrointestinal toxicity (immune-related colitis leading to diarrhea), hepatotoxicity (immune-related hepatitis causing elevated transaminases), and endocrine toxicity (hypothyroidism or hyperthyroidism, adrenal insufficiency, type 1 diabetes, etc.). In addition, relatively uncommon but serious immune-mediated adverse events may occur, such as pneumonitis (immune-related inflammation of the lungs leading to cough and dyspnea), nephritis (immune-related kidney injury leading to elevated serum creatinine), neurologic toxicities (peripheral neuropathy or central nervous system inflammation), hematologic abnormalities (e.g. autoimmune hemolytic anemia or thrombocytopenia), and others.
- **Hyperbaric Oxygen Therapy (HBOT):** Possible adverse reactions include middle ear barotrauma (ear pain, tympanic membrane injury), sinus or hollow-organ barotrauma, oxygen toxicity (extremely rare – for example, seizures can occur if high-pressure oxygen exposure is too prolonged), and claustrophobia, among others. However, HBOT is generally well tolerated, and the above reactions can usually be alleviated with appropriate intervention.

Investigators will pay special attention to these anticipated adverse events. If any occur, they will be managed without delay, and their severity will be assessed and reported according to the procedures described below.

3. Causality Assessment Between AE and Study Drug

For each adverse event, the investigator will evaluate the potential relationship to the study drug and record the rationale in the CRF. The assessment will be categorized as follows:

- **Definitely related:** The timing of the event strongly correlates with the study drug administration, and the clinical presentation aligns with the known adverse reaction profile of the drug. The reaction improves upon stopping the drug and reappears with re-challenge.
- **Highly likely related (Probable):** The event timing is consistent with dosing, and the type of reaction is in line with known effects of the study drug. There is no other more plausible explanation for the event; even without re-challenge, it is very likely drug-induced.
- **Possibly related:** The timing of the event is compatible with drug exposure, and the event could be due to the study drug, but it also might be attributable to the subject's underlying disease or other factors. Evidence is insufficient to be more certain.
- **Unlikely related:** The occurrence of the event has little temporal relationship with the drug administration, or the nature of the event does not fit the known adverse profile of the drug. It is more likely caused by the subject's underlying condition or other treatments.
- **Not related:** The adverse event is clearly caused by other factors and is not related to the study drug (for example, the event began before administering the study drug or can be definitively attributed to another cause).

The investigator's judgment will be used for classifying AEs in safety analyses. If the investigator is unsure of the causality, the event should be conservatively classified as "possibly related" to heighten vigilance regarding potential drug risks.

4. Adverse Event Severity Grading Criteria

Adverse event severity will be graded according to the National Cancer Institute Common Terminology Criteria for Adverse Events (NCI CTCAE) v5.0. If an adverse event is encountered that is not listed in CTCAE, the following grading descriptions can be referenced:

- **Grade 1 (Mild):** Mild symptoms; no impact on daily activities or function; tolerable without treatment.
- **Grade 2 (Moderate):** Moderate discomfort; some impact on daily activities, but the patient remains capable of self-care; may require some supportive care.
- **Grade 3 (Severe):** Severe symptoms; marked limitation in daily functioning, unable to carry out normal activities; usually requires active medical intervention (e.g. hospitalization or invasive intervention).
- **Grade 4 (Life-threatening):** Life-threatening consequences or urgent intervention required (e.g. shock, organ failure); immediate medical management is necessary.
- **Grade 5 (Death):** The adverse event results in death of the patient (directly or indirectly).

At each occurrence of an AE during the study, the severity should be graded according to the most severe intensity observed at that time. If the severity of the same event fluctuates over time, the highest grade attained should be recorded.

5. Definition of Serious Adverse Events (SAEs)

A serious adverse event is any untoward medical occurrence associated with the study (whether related to the investigational drug or not) that meets **at least one** of the following criteria:

- Results in death.
- Is life-threatening (i.e. the patient was at risk of death at the time of the event if no immediate intervention is provided).
- Requires inpatient hospitalization or prolongation of existing hospitalization.

- Results in persistent or significant disability/incapacity.
- Leads to a congenital anomaly or birth defect (if the subject was exposed to the investigational drug during pregnancy).
- **Other important medical events:** An event which, based on appropriate medical judgment, may not fall into the categories above but jeopardizes the patient's health and may require medical or surgical intervention to prevent one of the outcomes listed above.

Any adverse event that meets the above definition should be considered an SAE. For example, if a patient experiences a severe infusion reaction during treatment that requires urgent intervention, it should be reported as an SAE even if hospitalization is not needed. Likewise, an immune-related adverse event that necessitates long-term corticosteroid therapy should be evaluated for seriousness under these criteria.

6. Handling and Reporting of SAEs

Once an SAE is identified, the investigator must immediately take all necessary medical measures to ensure the safety of the subject and report the event as soon as possible. The investigator should notify the sponsor and relevant parties within 24 hours of becoming aware of the SAE. In accordance with regulatory requirements, all SAEs, regardless of attribution to the study drug, must be promptly reported to the study sponsor, the site's Ethics Committee, and the appropriate regulatory authorities. Reporting will be done in writing, for example by completing an SAE report form. (A template SAE report form is provided in Appendix 3 of this protocol.) The investigator should fill out the form completely and fax or email it to the designated contact. The report must include the subject's ID, the date of event onset, a detailed description of the event course, interventions taken, the outcome of the event, and the investigator's assessment of the event's relationship to the study drug. After the initial report, if there are further developments in the SAE, follow-up reports should be submitted in a timely manner until the event is resolved or stabilized. The Ethics Committee expects to receive an SAE report as soon as possible, generally an initial written report within 7 days. For SAEs that are related to the study and result in death or are life-threatening, the Ethics Committee should be informed within 24 hours (by phone or written communication). The investigator must keep copies of all SAE reports, and document the SAE in the CRF and the investigator's study file.

7. Criteria for Early Termination of the Trial

If any of the following situations occur during the study, the principal investigator and the sponsor will consider terminating the entire trial early to ensure participant safety:

- During the study treatment period, **unacceptable serious toxicity or a significant safety risk related to the trial treatment emerges** (for example, the incidence of death or life-threatening AEs significantly exceeds expectations), indicating that the risk of the trial therapy far outweighs its potential benefit.
- An **ethical review** concludes that continuing the trial is no longer scientifically meaningful or that the study hypothesis cannot be adequately tested (for instance, if the treatment efficacy is significantly worse than existing standard therapy, or if enrollment cannot be completed in a reasonable time frame).
- The **sponsor decides to terminate** the trial early (e.g. due to funding or drug supply issues), or a **regulatory authority/ethics committee mandates a halt** to the trial (e.g. due to discovery of major compliance or safety issues in the study).
- Other **force majeure factors** that make it impossible to continue the trial as planned (such as the investigational drug being withdrawn from the market, or the study site being unable to continue conducting the trial).

If a decision is made to terminate the trial early, the investigators should notify all participants and arrange necessary follow-up and appropriate alternative treatment. The research team should record the reasons and circumstances of the trial termination in detail and report them to the Ethics Committee and relevant regulatory authorities.

8. Other Safety Monitoring Measures

This study will implement a data and safety monitoring plan tailored to the risk profile of the trial. During the study, the investigator will periodically (for example, every six months) summarize all adverse events across participants and perform a cumulative review and analysis of safety data. If any abnormal safety signal is detected, it will be promptly discussed among the investigators, and necessary measures will be taken. The sponsor may appoint independent monitors to conduct regular on-site monitoring visits to ensure the accuracy of safety data and the timely reporting of adverse events. All these measures are intended to maximally protect the rights and well-being of the participants and to ensure the quality of the trial.

9. Dose Adjustments

9.1 Dose Adjustments for Chemotherapy Agents

If a toxicity is clearly attributable to one chemotherapeutic agent, reducing the dose of that single agent is acceptable. If the toxicity cannot be definitively linked to one agent (e.g. multiple agents may be contributing), then all suspect agents can be dose-reduced simultaneously. The maximum allowable interruption of chemotherapy is 6 weeks from the last dose. Before any chemotherapy cycle, if prior chemotherapy has caused hematologic toxicity, supportive treatment with colony-stimulating factors (e.g. G-CSF/GM-CSF) is permitted; however, prophylactic use of these growth factors **before the first dose** of chemotherapy is not allowed.

9.1.1 General Considerations

- Baseline body weight is used to calculate chemotherapy doses. If a subject's weight changes by more than ±10% from baseline, the doses should be adjusted accordingly. Weight changes of less than ±10% do not require dose modifications.
- If multiple toxicities occur at the same time with different severity grades, dose adjustments should be based on the highest observed grade.
- Prior to the start of each treatment cycle, the absolute neutrophil count (ANC) must be $\geq 1.5 \times 10^9$/L and the platelet count $\geq 75 \times 10^9$/L; otherwise, chemotherapy should be delayed. Based on the lowest platelet and neutrophil counts observed in the previous cycle, dose adjustments should be made at the start of the next cycle.
- If certain adverse effects are unlikely to progress to serious or life-threatening events (e.g. alopecia, taste changes, or nail changes), treatment can continue at the original dose level with no dose reduction or delay.
- If anemia (non-hemolytic) can be effectively managed with transfusions, dose reduction or delay of chemotherapy may not be necessary.
- If administration of any individual study drug is delayed by one day or more due to toxicity, then **all** study drugs in that cycle should be delayed by the same length of time. (Note: Scheduled tumor assessment time points will not be changed due to chemotherapy delays.)
- If a subject must delay the XELOX chemotherapy regimen for more than 3 weeks (i.e. >6 weeks from the start of the previous cycle) because of toxicity, the XELOX chemotherapy will be permanently discontinued due to unacceptable toxicity.
- If the investigator determines that a toxicity is caused by one specific drug in the regimen, that drug should be delayed or dose-reduced as needed (and the reduced dose will be used in subsequent cycles). If no contraindication exists, other chemotherapy drugs may continue on schedule.
- If continuing the study treatment is not in the subject's best interest, the investigator may decide to discontinue any individual chemotherapeutic agent or all chemotherapy agents in the regimen.
- The reason for any dose adjustment or delay, any supportive measures taken, and the outcome must be documented in the patient's records and recorded in the case report form.

9.1.2 Dose Adjustments for Hematologic Toxicity

For reference, the standard starting doses in this study are: oxaliplatin 130 mg/m² on Day 1; capecitabine 1,000 mg/m² orally twice daily on Days 1-14 of a 3-week cycle. Dose reduction levels are defined as: **Starting dose=100%**; **Level-1 (-25%):** oxaliplatin ≈100 mg/m², capecitabine ≈75% of original dose; **Level-2 (further reduction):** oxaliplatin ≈85 mg/m² (or discontinued), capecitabine ≈50% of original dose.

Table 1: Dose Adjustment Guidelines for Hematologic Toxicities

Hematologic Toxicity		Current Cycle Management	Resumption / Next Cycle Dose	Upon Recurrence
**Neutropenia**	*Pre-cycle counts below threshold:* ANC < 1.5 × 10^9/L or PLT < 100 × 10^9/L.	Delay chemotherapy by 1 week and recheck counts (maximum delay 2 weeks).	If counts recover within 7 days → continue at original dose; ~14-day delay → resume at Level -1; ≥21-day delay without recovery → consider discontinuing treatment.	—
	Grade 2 (ANC 1.0–<1.5 × 10^9/L), no fever.	May continue treatment or briefly pause, per institutional guidelines.	After counts meet criteria → continue at original dose.	If recurrent or prolonged, consider Level -1.
	Grade 3 (ANC 0.5–<1.0 × 10^9/L), no fever.	Delay chemotherapy until ANC ≥ 1.5 × 10^9/L.	After recovery → resume at original dose or Level -1 (per clinical judgment).	Recurrence → implement Level -1.
	Grade 4 (ANC < 0.5 × 10^9/L) **or** Febrile Neutropenia (FN).	Delay until ANC ≥ 1.5 × 10^9/L & PLT ≥ 75 × 10^9/L; manage any infection.	Next cycle: resume at Level -1.	Recurrence → Level -2 or discontinue chemotherapy; consider G-CSF secondary prophylaxis.
**Thrombocytopenia**	*Grade 1* (PLT 75–<100 × 10^9/L).	Generally delay treatment; if clinically stable, some patients may continue.	After PLT recovers to ≥ 100 × 10^9/L (or institutional threshold) → continue at original dose, or consider Level -1 for high-risk cases.	Recurrence → implement Level -1.
	Grade 2 (PLT 50–<75 × 10^9/L).	Delay until PLT ≥ 75–100 × 10^9/L.	Resume at Level -1.	Recurrence → Level -2 or discontinue.
	Grade 3–4 (PLT < 50 × 10^9/L).	Delay until PLT ≥ 75–100 × 10^9/L.	Resume at Level -1.	Recurrence → Level -2 or discontinue.
**Anemia**	*Anemia (symptomatic or Hb < 8 g/dL).*	Provide supportive care (e.g. transfusion); hold chemotherapy if necessary.	After recovery, manage per clinical assessment (generally no fixed dose reduction solely for anemia).	Recurrence → individualize management.

Notes:
a. Neutrophil and platelet toxicity grading thresholds are based on CTCAE v5.0 criteria.
b. Following any episode of febrile neutropenia or Grade 4 neutropenia, or a platelet count <50 × 10^9/L,

chemotherapy should be delayed until recovery, and a 25% dose reduction of both drugs should be considered. If platelet count is 75–100 × 10^9/L, treatment may continue in select cases; otherwise, delay until adequate recovery.
c. The thresholds for resuming treatment and the dose reduction after severe hematologic toxicity should follow the recommendations in the oxaliplatin prescribing information.
d. Examples of initial count thresholds, delay durations, and dose reduction strategies (as well as anemia management) can be referenced from the NHS CAPOX standard operating procedure (SOP).
e. If during a cycle of therapy the lab results show ANC < 1.0 × 10^9/L or PLT < 75 × 10^9/L while the patient is taking capecitabine, capecitabine should be interrupted immediately. After recovery, restart capecitabine at a reduced dose according to the grade of toxicity.
f. G-CSF: For patients who have experienced FN or persistent severe neutropenia in prior cycles, secondary prophylaxis with G-CSF may be used in subsequent cycles.

9.1.3 Dose Adjustments for Non-hematologic Toxicity

Table 2: Oxaliplatin Dose Adjustment for Non-hematologic Toxicities

Toxicity	Grade / Criteria	Immediate Management	Subsequent Adjustment
**Hypersensitivity / Allergic Reaction**	**Grade 1–2** (e.g. rash, pruritus, mild wheezing)	Interrupt oxaliplatin infusion; administer antihistamines and/or bronchodilators. After symptom relief, resume infusion at a slower rate.	Next cycle: premedicate (H1 blocker ± H2 blocker ± corticosteroid) and infuse at a slower rate. If Grade 2 reaction recurs → consider discontinuation of oxaliplatin or a desensitization protocol.
	**Grade 3–4** (e.g. hypotension or O_2 desaturation, bronchospasm, anaphylaxis)	Immediately stop infusion and initiate emergency management.	Permanently discontinue oxaliplatin.
**Suspected Interstitial Lung Disease (ILD) / Pulmonary Toxicity**	Any suspicion of ILD or serious lung toxicity	Hold oxaliplatin and conduct thorough evaluation (imaging and pulmonary consultation).	If evaluation confirms **no ILD** and symptoms ≤ Grade 1 → oxaliplatin may be resumed at the same dose or at Level -1 (per clinical judgment). If ILD is confirmed → permanently discontinue oxaliplatin.
**Nausea/Vomiting** (despite triple/quadruple antiemetic prophylaxis)	**Grade 3–4** (persistent or recurrent severe nausea/vomiting)	Delay chemotherapy until symptoms improve to ≤ Grade 1; optimize antiemetic regimen.	For the first occurrence, chemotherapy may continue at the same dose or with a Level -1 dose reduction. If severe nausea/vomiting recurs → reduce to Level -2 or consider omitting oxaliplatin from the regimen.
**Diarrhea / Oral Mucositis**	**Grade 3–4**	Interrupt capecitabine and provide supportive care (e.g. rehydration, anti-diarrheals). If necessary, delay oxaliplatin in the same	After recovery to ≤ Grade 1: oxaliplatin can typically be resumed at the original dose. If Grade ≥3 diarrhea/mucositis recurs, or if associated with dehydration or renal impairment

Toxicity	Grade / Criteria	Immediate Management	Subsequent Adjustment
		cycle.	→ resume oxaliplatin at a Level -1 reduced dose.
**Pancreatitis**	**Grade 3–4**	Hold chemotherapy and investigate the cause of pancreatitis.	If pancreatitis is confirmed to be drug-related → permanently discontinue oxaliplatin. If pancreatitis is unrelated to the drug and the patient fully recovers → oxaliplatin may be resumed at the original dose.
**Skin Toxicity** (e.g. Hand–Foot Syndrome)	**Grade 3–4**	Interrupt capecitabine and provide supportive dermatologic treatment; consider delaying oxaliplatin during this cycle.	After recovery to ≤ Grade 1: oxaliplatin may be continued without dose reduction (no need to reduce oxaliplatin for skin toxicity once resolved).

Table 3: Oxaliplatin Dose Adjustment for Neurotoxicity

Neurotoxicity (Oxaliplatin-Induced)	Duration / Scenario	Management During Current Cycle	Dose Adjustment for Next Cycles
**Peripheral sensory neuropathy (paresthesia/dysesthesia) not affecting function** (Grade 1)	Any duration	Continue oxaliplatin.	No dose change (continue at current dose).
**Peripheral neuropathy with pain or mild functional impairment** (Grade 2)	≤ 7 days duration	Continue oxaliplatin.	No dose change (continue at current dose).
	> 7 days duration, or recurrent episodes	Continue oxaliplatin or consider a brief pause until improvement.	Reduce to Level -1 dose for subsequent cycles.
**Neuropathy with obvious impact on daily living** (Grade 3)	Any duration	Hold oxaliplatin until symptoms improve to ≤ Grade 1.	After recovery, resume oxaliplatin at a reduced dose (Level -1 or Level -2), or discontinue oxaliplatin.
**Severe neuropathy – disabling or life-threatening** (Grade 4)	Any duration	Permanently discontinue oxaliplatin.	— (oxaliplatin stopped)
**Acute pharyngolaryngeal dysesthesia (PLD)** – *sensation of throat tightening or inability to breathe, occurring during or within ≤2 hours after infusion, often cold-induced*	First occurrence	Interrupt oxaliplatin infusion; administer warm oxygen and sedatives as needed. After symptoms resolve, resume infusion at **1/3** of the original rate.	For subsequent cycles: extend infusion duration to ~6 hours or significantly slow the infusion rate, and strictly avoid cold exposure for 48 hours post-infusion. Dose reduction is generally not required for isolated PLD. If PLD recurs and significantly affects therapy → reduce oxaliplatin dose by one level (Level -1).

9.2 Dose Adjustments for Sintilimab

Sintilimab (an anti–PD-1 monoclonal antibody) can cause various immune-related adverse events. The most common toxicities involve the skin and mucous membranes, the gastrointestinal tract (colitis/diarrhea), the liver (hepatitis), and the endocrine system. However, immune-related adverse events can also affect other organ systems such as the thyroid, lungs, kidneys, eyes, nervous system, or blood. The severity of immune-related adverse events should be graded according to the immune checkpoint inhibitor criteria in CTCAE v5.0.

Table 4: Guidelines for Temporarily Withholding or Permanently Discontinuing Sintilimab

Adverse Event (Related to Sintilimab)	Severity	Action
**Pneumonitis**	Grade 2 pneumonitis	Temporarily withhold (a)
	Recurrent Grade 2 pneumonitis, or Grade 3–4 pneumonitis	Permanently discontinue
**Diarrhea / Colitis**	Grade 2 or 3 diarrhea or colitis	Temporarily withhold (a)
	Grade 4 diarrhea or colitis	Permanently discontinue
**Dermatitis**	Grade 3 dermatitis	Temporarily withhold (a)
	Grade 4 dermatitis	Permanently discontinue
**Hepatitis** (immune-mediated)	For patients with baseline ALT/AST/TBIL in normal range: Grade 2 elevation of ALT or AST or total bilirubin; For patients with baseline ALT/AST/TBIL above ULN: ALT/AST/TBIL increase ≥50% from baseline (meeting Grade 2 criteria) **and** duration < 7 days.	Temporarily withhold (a)
	For patients with baseline ALT/AST/TBIL in normal range: Grade 3 or 4 elevation of ALT or AST or total bilirubin; For patients with baseline ALT/AST/TBIL above ULN: ALT/AST/TBIL increase ≥50% from baseline (meeting Grade 3–4 criteria) **and** duration ≥ 7 days.	Permanently discontinue
**Hypophysitis** (pituitary inflammation)	Grade 2 hypophysitis	Temporarily withhold (b)
	Grade 3 or 4 hypophysitis	Permanently discontinue
**Adrenal Insufficiency**	Grade 2 adrenal insufficiency	Temporarily withhold (b)
	Grade 3 or 4 adrenal insufficiency	Permanently discontinue
**Hyperthyroidism**	Grade 3 or 4 hyperthyroidism	Permanently discontinue
**Type 1 Diabetes** (immune-mediated)	Grade 3 hyperglycemia	Temporarily withhold (b)
	Grade 4 hyperglycemia	Permanently

Adverse Event (Related to Sintilimab)	Severity	Action
		discontinue
**Renal Impairment** (nephritis)	Grade 2 or 3 elevation in serum creatinine (Cr)	Temporarily withhold (a)
	Grade 4 elevation in serum creatinine	Permanently discontinue
**Neurologic Toxicity**	Grade 2 neurologic toxicity	Temporarily withhold (a)
	Grade 3 or 4 neurologic toxicity	Permanently discontinue
**Other AEs**	Any other Grade 3 AE – first occurrence	Temporarily withhold (a)
	Same Grade 3 AE – second occurrence	Permanently discontinue
	Grade 3 AE that does not improve to Grade 0–2 within 7 days, **or** does not recover to Grade 0–1 within 14 days	Permanently discontinue
	Grade 4 AE	Permanently discontinue (c)

Notes:
a. Sintilimab may be resumed only after the toxicity has improved to ≤ Grade 1 and the patient has been tapered off systemic corticosteroids to ≤ 10 mg/day prednisone (or equivalent) **or discontinued**, with no ongoing need for other immunosuppressive therapy. These conditions must be met before considering reintroduction of sintilimab (resumption after a prior Grade 3 event should be approached with particular caution).
b. Once the adverse event is clinically stable or the patient's metabolic state has normalized, immunotherapy can be continued or resumed without strictly requiring corticosteroid taper to ≤ 10 mg/day prednisone equivalent (the criterion in note a). In other words, for certain endocrine events that have stabilized (e.g. adrenal insufficiency or Type 1 diabetes), sintilimab can be resumed at the physician's discretion even if the patient requires ongoing hormone replacement above the 10 mg/day threshold.
c. **Permanent discontinuation** of sintilimab is required for any Grade 4 immune-related adverse event (irAE), except for endocrine toxicities that can be managed with hormone replacement therapy. Permanent discontinuation is also required for any life-threatening or high-risk organ involvement (e.g. myocarditis; neurologic toxicity such as encephalitis, Guillain–Barré syndrome, or myasthenia gravis; Grade 3–4 immune-related pneumonitis); for recurrence of the same irAE at ≥ Grade 3 severity; or if an irAE does not improve within 48–72 hours of holding therapy and administering adequate steroids (or if second-line immunosuppressive therapy is needed). Additionally, if > 10 mg/day prednisone (or equivalent) is required for > 12 weeks to control an immune-related adverse event, sintilimab should be permanently discontinued.

9.3 Dose Adjustments for HBOT

During hyperbaric oxygen therapy (HBOT), if an adverse reaction occurs, the investigator should manage it by either **temporarily pausing** the treatment or **permanently discontinuing HBOT**, depending on the type and severity of the toxicity. All adverse events will be promptly evaluated, treated, and reported to the Medical Safety Team (MST). If HBOT is paused or permanently stopped due to an adverse event, this must be documented in detail and included in the final data analysis and safety report.

Table 5: Guidelines for Temporarily Pausing or Permanently Discontinuing HBOT

Adverse Event Type	Severity / Criteria	Immediate Management	Subsequent Action
**Oxygen Toxicity** (CNS / Pulmonary)	**Mild (Grade 1–2):** Nausea, dizziness, slight facial or eyelid twitching, tinnitus, visual flashes, or irritative cough (symptoms present but tolerable).	Immediately initiate an air break (breathing room air) for 5–10 minutes. After symptoms resolve, continue the HBOT session.	No change to HBOT protocol is needed **(a)**.
	**Moderate or Recurrent:** Symptoms above persist or significantly impede patient cooperation (e.g. occurs in ≥ 2 sessions).	Terminate the HBOT session for that day; evaluate the patient and provide symptomatic treatment as needed.	Once symptoms have fully resolved for ≥ 24 hours, resume HBOT with the original protocol. If oxygen toxicity occurs again → **permanently discontinue HBOT**.
	**Severe (Grade 3–4 or seizure):** Convulsion or other life-threatening signs of oxygen toxicity.	Immediately remove the patient from breathing 100% O_2 (switch to air), and begin decompression of the chamber. Ensure the airway is secure; administer a benzodiazepine to control seizures if necessary. Once the patient is stabilized, abort the treatment and emergently transfer for medical care.	**Permanently discontinue HBOT.**
**Middle Ear Barotrauma**	**Mild:** Ear fullness, tinnitus, mild ear pain, **no** otoscopic signs of bleeding.	Pause the session; administer a nasal decongestant and perform pressure equalization techniques (e.g. Valsalva) **(b)**.	Resume HBOT after symptoms fully resolve. If barotrauma recurs → obtain an **ENT** evaluation **(c)** before continuing further treatments.
	**Moderate:** Blood in the middle ear (hemotympanum), significant ear pain, or noticeable hearing loss.	Stop HBOT for the current treatment cycle and refer the patient to **ENT** for evaluation and management.	After recovery and clearance by ENT, HBOT may be resumed if appropriate. If needed, perform tympanostomy or place ventilation tubes before resuming HBOT. Patients with frequent recurrence of ear barotrauma should **permanently discontinue HBOT**.
	**Severe:** Tympanic membrane perforation.	Immediately discontinue HBOT; obtain emergency ENT care for perforation.	**Permanently discontinue HBOT.**
**Sinus or Dental Barotrauma**	**Mild–Moderate:** Sinus pressure or pain	Pause the session; administer nasal decongestants or	Resume HBOT only after symptoms resolve and the

Adverse Event Type	Severity / Criteria	Immediate Management	Subsequent Action
	(forehead/face), tooth pain, tenderness over sinuses.	provide dental treatment as appropriate.	patient can equalize sinus pressure. If barotrauma recurs in ≥ 2 sessions → discontinue HBOT for the remainder of the cycle.
**Pulmonary Barotrauma** (e.g. pneumothorax or mediastinal emphysema)	**Any suspected or confirmed case:** Signs of pneumothorax or other air embolism syndromes.	Immediately halt HBOT; administer high-flow oxygen; perform prompt imaging (e.g. chest X-ray) and involve thoracic surgery for intervention.	If a pneumothorax is confirmed → **permanently discontinue HBOT (d)**.
**Claustrophobia / Anxiety**	**Mild:** Feelings of claustrophobia or anxiety without panic.	If needed, stop the session and remove the patient from the chamber. Provide reassurance, relaxation techniques, and psychological support.	After patient is calmed and evaluated, HBOT can be resumed. If necessary, consider a short-acting anxiolytic before future sessions **(f)**.
	**Moderate–Severe or Repeated Episodes:** Intense panic, anxiety, or claustrophobia that interferes with treatment, or recurrent mild episodes.	Discontinue HBOT for the current cycle; refer the patient for psychological or psychiatric evaluation if appropriate.	If the patient is unable to tolerate HBOT despite interventions (repeated episodes) → **permanently discontinue HBOT**.
**Hypoglycemia** (in diabetic patients)	**Any episode:** Blood glucose < 3.9 mmol/L (70 mg/dL) or symptomatic hypoglycemia; or blood glucose 3.9–<6.0 mmol/L (108–<108 mg/dL) with risk of dropping further.	If glucose < 3.9 mmol/L or patient has symptoms: immediately administer a fast-acting carbohydrate (oral glucose), monitor the patient, and postpone HBOT. If pre-HBOT glucose is 3.9–<6.0 mmol/L, give a carbohydrate snack and recheck glucose before proceeding.	Check finger-stick blood glucose before and after each HBOT session; maintain pre-HBOT glucose in the 6.0–13.9 mmol/L (108–250 mg/dL) target range. If hypoglycemia occurs in ≥ 2 consecutive sessions → hold further HBOT in the cycle and consult an endocrinologist **(e)**.
**Vision Changes** (oxygen-induced myopia)	**Mild blurred vision or myopic shift:** Difficulty focusing or noted myopia increase during HBOT course.	Continue HBOT – no acute intervention required aside from observation. Document the vision change and follow up over time.	Vision changes from HBOT are typically reversible within a few weeks after therapy completion. If visual changes significantly impair daily function, the Principal Investigator should determine whether to temporarily pause HBOT until vision stabilizes.

Notes:

a. In this study, the approach to HBOT adverse effects is to **pause treatment or permanently terminate HBOT** as needed; there is no adjustment to the pressure or oxygen dose used in HBOT sessions.

b. **Ear pressure equilibration and decongestion:** Before HBOT sessions, train patients in techniques to equalize ear pressure (e.g. Valsalva maneuver, swallowing, jaw movements). A short course of nasal decongestant (such as saline spray or a brief use of topical vasoconstrictive drops) may be used to help with Eustachian tube opening. Use vasoconstrictor decongestants with caution in patients with hypertension or glaucoma.

c. **ENT evaluation:** Indications for referral to an Ear, Nose, and Throat (ENT) specialist (otorhinolaryngologist) include persistent inability to equalize ear pressure, recurrent middle ear barotrauma, or need for procedures such as tympanostomy or pressure-equalization tube placement. HBOT should not be resumed until any barotrauma injury has healed or been managed appropriately.

d. **Pneumothorax:** An untreated pneumothorax is an absolute contraindication for HBOT. If pneumothorax is suspected during HBOT, obtain immediate imaging (e.g. chest X-ray). If a pneumothorax is confirmed, it must be managed with chest tube decompression; the patient should not continue in this HBOT study (a confirmed pneumothorax warrants permanent discontinuation of HBOT in the trial). Any future consideration of HBOT (outside the study) should only occur after full recovery and with appropriate precautions.

e. **Glucose management:** Before HBOT, a target blood glucose of 6.0–13.9 mmol/L (108–250 mg/dL) is recommended. If blood glucose is < 3.9 mmol/L or > 16.7 mmol/L (300 mg/dL) with positive urinary ketones, HBOT should be postponed. Manage hypoglycemia using the "15–15 rule" (ingest 15 grams of fast-acting carbohydrate and recheck glucose after 15 minutes, repeating as needed) and verify that glucose has normalized before starting or resuming HBOT. Always re-check blood glucose after HBOT sessions as well, since HBOT can affect glucose levels.

f. **Sedation for anxiety:** If necessary, a small dose of a short-acting benzodiazepine can be administered 30–60 minutes before an HBOT session to alleviate anxiety or claustrophobia. Use sedation cautiously, especially in elderly patients or those with COPD, as sedatives can depress ventilation and potentially affect pressure equilibration in the ears. Patients who receive a sedative must be monitored and instructed not to drive or operate machinery for the remainder of the day after the HBOT session.
